# A fluorescently-tagged tick kinin neuropeptide triggers peristalsis and labels tick midgut muscles

**DOI:** 10.1038/s41598-024-61570-w

**Published:** 2024-05-13

**Authors:** Jonathan R. Hernandez, Caixing Xiong, Patricia V. Pietrantonio

**Affiliations:** Department of Entomology, Texas A&M University, College Station, TX 77843-2475 USA

**Keywords:** G protein-coupled receptor (GPCR), Muscle contraction, Tick kinin, Tick physiology, Midgut, Fluorescently labeled peptide, Confocal microscopy, Peptides, Microscopy, Fluorescence imaging, Entomology, Biological techniques, Physiology, Endocrinology

## Abstract

Ticks are blood-feeding arthropods that require heme for their successful reproduction. During feeding they also acquire pathogens that are subsequently transmitted to humans, wildlife and/or livestock. Understanding the regulation of tick midgut is important for blood meal digestion, heme and nutrient absorption processes and for aspects of pathogen biology in the host. We previously demonstrated the activity of tick kinins on the cognate G protein-coupled receptor. Herein we uncovered the physiological role of the kinin receptor in the tick midgut. A fluorescently-labeled kinin peptide with the endogenous kinin 8 sequence (TMR-RK8), identical in the ticks *Rhipicephalus microplus* and *R. sanguineus*, activated and labeled the recombinant *R. microplus* receptor expressed in CHO-K1 cells. When applied to the live midgut the TMR-RK8 labeled the kinin receptor in muscles while the labeled peptide with the scrambled-sequence of kinin 8 (TMR-Scrambled) did not. The unlabeled kinin 8 peptide competed TMR-RK8, decreasing confocal microscopy signal intensity, indicating TMR-RK8 specificity to muscles. TMR-RK8 was active, inducing significant midgut peristalsis that was video-recorded and evaluated with video tracking software. The TMR-Scrambled peptide used as a negative control did not elicit peristalsis. The myotropic function of kinins in eliciting tick midgut peristalsis was established.

## Introduction

Ticks are blood-feeding arthropods that during the feeding process acquire and transmit a wide range of pathogens causative of diseases to humans, livestock, pets, and wildlife^1^. Depending on their species, ixodid ticks can feed while attached to hosts for several days to weeks. Blood-feeding in ixodid ticks involves two major phases: the slow feeding phase and the rapid feeding phase. The slow phase (4–9 days) is characterized by a ten-fold increase in weight and the beginning of cuticle synthesis of the tick integument that accommodates the increasing blood volume and, in the midgut, the first continuous digestion proceeds^2–4^. It is during the slow feeding phase that pathogen transmission occurs^5^. During the rapid phase (~ 1 day) ticks feed to repletion (~ 100 times their unfed body mass) enabling them to reach the critical weight required for drop-off from the host and vitellogenesis; only mated females can engorge rapidly^6^. During the rapid feeding phase intracellular digestion is reduced in the midgut and is when vitellogenin-producing basophilic cells begin to form and vitellogenin synthesis and vitellogenesis begins^4,7^. After dropping off the host, the preoviposition phase coincides with the second discontinuous digestion. The midgut is critical for tick reproduction as together with the fat body synthesizes vitellogenin(s)^8^. While the role of ecdysteroids in vitellogenesis has been established^9–11^, there is less information on other regulators such as neuropeptides.

G protein-coupled receptors (GPCRs) are a large superfamily of membrane proteins that play crucial roles in cellular signaling, regulating various physiological processes in both vertebrates and invertebrates. Insect (leuco)kinin receptors are Family A GPCRs that are involved in a wide range of physiological processes after activation by kinin neuropeptides (first named leucokinins because of their discovery in the roach *Leucophaea maderae*^12^). Insect kinin peptides exert myostimulatory action on visceral muscles^13–15^, and are involved in regulation of diuresis^16,17^ and feeding^18–20^, endocrine regulation^21^, sugar taste responses^22^, pre-ecdysis behavior^23^, as well as tracheal air clearance during ecdysis^24^ and other functions^25^. Furthermore, GPCRs have been proposed as unexploited targets for insecticides^26^ and this has been demonstrated with insect kinin analogs being deadly to aphids and considered eco-friendly insecticides with minimal toxicity to non-target organisms^27,28^. In ticks, the leucokinin-like peptide receptor, hereafter referred to as the tick kinin receptor, also plays a crucial role in regulating nutrition-dependent fecundity^29^.

The midgut is the largest organ within the body of the tick^30^. This spider-shaped structure spans across most of the body cavity and is comprised of a central stomach and several caeca^31^. The wall of the midgut is composed of an inner epithelium and thin outer layer of smooth muscle cells^30,32^. It functions to store and digest a blood meal from a vertebrate host^33–35^. Unlike blood-feeding insects, ticks exhibit almost entirely intracellular digestion within the midgut epithelial cells, contributing to the gradual and prolonged digestion of the consumed blood that remains in the midgut lumen. Thus, the midgut also acts as a food storage reservoir, allowing ticks to store undigested blood for extended periods, contributing to their ability to endure prolonged fasting. Furthermore, the midgut plays a pivotal role in vitellogenin synthesis and its incorporation into oocytes while carrying heme, which is a crucial step for successful reproduction^4,11,36–38^. The tick midgut also serves as the primary site of tick-pathogen interactions and can be colonized by pathogens, symbionts, and commensals which affects several midgut metabolic pathways such as those for glycolysis, gluconeogenesis, lipids, redox and amino acids^39–42^. Given the multifaceted functions of the gut in arthropods, it is unsurprising that signaling molecules play a pivotal role in regulating diverse processes, ranging from digestive functions and feeding behaviors to immune responses^43^. Thus, our understanding of tick midgut physiology is paramount for developing effective control strategies, preventing disease transmission, and exploring intervention methods that target key physiological processes. The midgut's complex network of muscles plays a critical role in facilitating the movement of ingested blood, mixing digestive enzymes, and maintaining peristaltic contractions essential for efficient blood meal processing^44^. In *R. sanguineus*, Lees et al.^45^ demonstrated the transcript expression of the tick kinin receptor in the gut, synganglion, salivary glands, Malpighian tubules and oviduct of partially fed females using the 3′ end of the gene sequence encompassing 83 aa and 663 bp (contig 7803_E3, GQ215233) that had 97% identity to the homologous *R. microplus* leucokinin-like peptide receptor (GenBank accession number: AAF72891.1)^46,47^.

Here we document the precise localization and functional relevance of the kinin receptor in the midgut of *R. sanguineus* ticks, a species known for its veterinary and medical importance. To achieve this, we visualized the receptor's distribution in the midgut and assessed its impact on midgut muscle contractions. This study not only enhances our understanding of tick physiology but also holds the potential for the development of innovative tick control methods, making it a significant area of research in the context of tick-borne disease management.

## Materials and methods

### Ticks

Unfed female *Rhipicephalus sanguineus* (Acari: Ixodidae) ticks were obtained from Ecto Services, Inc. (Henderson, NC, USA). Females were kept in polypropylene tubes (6 cm × 2.5 cm) sealed with a small piece of mesh (4 × 4 cm). Tubes were placed in a closed glass chamber prepared using a heavy-duty glass dome desiccator with a Porcelain Plate (6″/150 mm) and maintained at 97% relative humidity with a 99% saturated potassium sulfate solution below the plate. Ticks were kept at 26.5 ± 1 °C, in a 16:8 h (light: dark) photoperiod inside a Percival Incubator. Only unfed females aged 3 to 8 days-old were used for experiments. Older ticks engorged with blood were not used because if the dark, red blood would leak from the gut it would interfere with video tracking software and fluorescent labeling.

### Reagents and peptides

Chemical reagents were purchased from: Sigma (St. Louis, MO, USA) for potassium sulfate, KCl, MgCl_2_, HEPES; EMS Science (Gibbstown, NJ) for CaCl_2_ and NaCO_3_; or Macron (Central Valley, PA) for NaCl.

Three peptides were used in this study. These included: 1) the ligand for the endogenous tick kinin receptor from *Rhipicephalus sanguineus*, Rhisa-kinin 8 peptide of sequence GTGEDQAFSPWGamide (Rhisa-K-8; RK8; of identical sequence to Rhimi-K-8 in *Rhipicephalus microplus*)^48^, 2) the same peptide but fluorescently labeled at the N-terminus with tetramethylrhodamine (TMR) a red fluorescent dye, designated TMR-Rhisa-K-8 (TMR-RK8), and 3) a labeled peptide synthesized with the scrambled sequence of the kinin ligand, designated TMR-Scrambled. The sequence of TMR-RK8 was TMR-**C**-GTGEDQAFSPWGamide). The TMR-Scrambled kinin (TMR-**C**-GTQWGPGFEADSamide) served as a negative control for tissue labeling. All peptides were custom-synthesized and purchased from GenScript (Piscataway, NJ, USA) with a purity of ≥ 95% and the cysteine in bold in both sequences was added to attach the label. Experiments were replicated with independently synthesized batches (4 mg each) of labeled and unlabeled peptides, for reproducibility. Unlabeled RK8 was used as a competitive ligand to verify target-specific binding of the labeled TMR-RK8 (see *Ligand Competition Assay*). For cell assays, the peptide stock solutions (100 µM) were made in 1% dimethyl sulfoxide (DMSO) in Hank’s balanced salt solution (HBSS, Thermo Fisher, Waltham, USA). For fluorescent cell labeling, peptide working solutions (10 µM) were prepared in HBSS from stock solutions. For assays on the midgut, peptides were solubilized and diluted in a tick physiological saline consisting of 140 mM NaCl, 5 mM KCl, 1 mM MgCl_2_, 5 mM CaCl_2,_ 4 mM NaHCO_3_, and 5 mM HEPES, pH 7.2^49^.

### Cell culture

The tick kinin receptor is stably expressed in the recombinant CHO-K1 cell line designated BMLK3 (*Boophilus microplus* leucokinin receptor 3) that was constructed and selected as described by Holmes, et al.^47^. The CHO-K1 cells had been obtained from the American Type Culture Collection (ATCC) for transfection with the expression construct. This cell line has been kept in the laboratory since 2003. Cells that were transfected only with an empty plasmid are referred to as “vector-only” (V/O) cells and were used as a negative control. Before all experiments, the BMLK3 cell line or V/O cells were separately cultured in T-75 flasks with selective medium (F-12 K medium containing 10% FBS and 800 μgmL^−1^ of G418 sulfate) for one to two passages in a humidified incubator at 37 °C and 5% CO_2_.

### BLAST analyses of tick kinin receptor

To verify the similarity of the *Rhipicephalus sanguineus* kinin receptor to those from other tick species, we conducted a protein–protein BLAST (blastp) search of the ‘Non-redundant protein sequences (nr)’ and ‘Transcriptome Shotgun Assembly proteins (tsa_nr)’ databases against Acari (taxid: 6933) in NCBI using the *R. microplus* kinin receptor deduced amino acid sequence (GenBank accession number AAF72891.1) as the query sequence. The predicted protein sequence AAF72891.1 corresponds to the *R. microplus* kinin receptor mRNA sequence (AF228521.1) reported by Holmes, et al.^46^.

### Cellular end-point fluorescence calcium mobilization assay

A fluorescence-based intracellular calcium mobilization end-point assay described by Xiong, et al.^50^ was used to confirm the activity of the TMR-labeled RK8 peptide on a cell line expressing the tick kinin receptor. For this, BMLK3 and V/O cells were cultured in F-12 K medium (1% FBS and 400 μgmL^−1^- of G418 Sulfate). The cells were seeded in a 96-well plate with 20,000 cells per well and incubated overnight at 37 °C and 5% CO_2_. The next morning, cells were prepared for the assay following the manufacturer’s instructions for the Fluo-8 Calcium Flux Assay Kit – NoWash (Abcam, Cambridge, United Kingdom). The F-12 K medium in the plate was removed by inverting the plate and gently blotting on paper towels. The media was then replaced with 100 µL of Fluo-8 AM loading dye (1X). The plate was incubated at 37 °C under 5% CO_2_ for 30 min, then equilibrated at room temperature in the dark for an additional 30 min. The calcium fluorescence signal was detected by a CLARIOstar (BMG LABTECH, Chicago, IL, United States) plate reader set to read at the fluorescence plate-mode with excitation and emission wavelengths of 490 and 525 nm at 29 °C. Calcium responses were read immediately after the addition of 10 µL of either TMR-RK8, unlabeled RK8, or blank solvent (1% DMSO in HBSS). Dose–response curves for the BMLK3 cells were obtained by testing four different concentrations of either the TMR-RK8 or unlabeled RK8, ranging from 10 nM to 10 μM and the blank solvent. V/O cells were tested with 10 μM of either TMR-RK8 or unlabeled RK8, as well as the blank solvent. Cellular responses were recorded for a total of 60 s with 1 s intervals and were represented by the average fluorescence units recorded. Each treatment was performed with 4 technical replicates.

### Visualization of the *R. microplus* recombinant kinin receptor on live BMLK3 cells with fluorescently labeled kinin peptide

The BMLK3 and V/O cells (5000–10,000 cells) were seeded separately in 200 µL selective media onto the two chambers of the cover glass (Lab-Tek Chambered Coverglass, 4.2 cm^2^ area, #1.0 German borosilicate glass, sterile, Cat # 155,380, Thermo Fisher Scientific Inc., Walthams, MA) and grown in the incubator overnight at 37 °C and 5% CO_2_. The next day, cells would be ready for fluorescent labeling: the selective media was replaced with 200 µL HBSS to rinse the cells. Then the HBSS was removed and 200 µL of 10 µM of either TMR-Scrambled or TMR-RK8 were added to the tissue and incubated for 1 min. Cells were rinsed twice with 200 µL HBSS which was removed, and finally added 200 µL of fresh HBSS. The signal was verified and imaged using the Leica SP8 inverted confocal microscope (Leica Camera AG, Wetzlar, Germany) with the TMR (red, fluorescence excitation lasers at Ex 542 nm, Em 572 – 759 nm for TMR peptides) and DIC channels at 20×*g* at 0.96 µm steps. Images were analyzed and computer-colored using Fiji/ImageJ (National Institutes of Health, Bethesda, MD). Maximum intensity projections and videos of the z-stacks were then produced.

### Midgut dissection

Individual female ticks were immobilized on a Petri dish filled with wax by submerging their legs in melted wax heated with a soldering pencil (Electronic Control Soldering Station EC 1000, Weller, Apex, North Carolina, USA). Females were dissected under physiological saline following the protocol described by Tidwell et al.^51^. The tick was cut along the edge of the scutum/alloscutum using a stainless steel #12 scalpel blade (Integra Miltex, York, PA, USA), after which the scutum was removed with a pair of fine forceps. To expose the midgut for removal, the tracheae, Malpighian tubules, ovaries, rectal sac, and synganglion were removed with fine forceps in that order. The midgut was then carefully removed and placed into 200 µL of fresh tick saline (See Reagents and Peptides) on a single gasketed incubation chamber (500 µL, Size 22 × 40 mm, Electron Microscopy Sciences, Hatfield, PA). The midguts were then rinsed three times with the physiological tick saline for subsequent fluorescent labeling or activity experiments.

### Fluorescent labeling of live tissues

To determine the location of the tick kinin receptor in the midgut of ticks, a commercially synthesized Rhisa-kinin peptide 8^52^ conjugated with a stable, red fluorophore (TMR-RK8) or a similarly labeled peptide of scrambled sequence (TMR-Scrambled) were applied to freshly dissected midguts ex vivo. For this, midguts of unfed adult females were incubated in ice-cold saline for 10 min. The saline was removed before incubation in 200 µL of 4’,6-diamidino-2-phenylindole dihydrochloride (DAPI, Sigma, 1 µg/mL in saline) for 1 min and rinsed with saline (200 µL) once. After saline removal, the midgut was incubated for 1 min with 200 µL of 10 µM of either TMR-Scrambled kinin or TMR-RK8, then the labeled peptide solution was removed by pipette and the midgut was rinsed twice with 200 µL saline. The saline was removed and Alexa Fluor 488 Phalloidin (200 µL) (Cell Signaling Technology, Danvers, MA) was added to the midgut and incubated for 15 min. After removal of the phalloidin solution, 200 µL of saline were added and a micro cover slip (24 × 60 mm, VWR, Radnor, PA) was placed over the gasketed portion of the incubation chamber; the chamber was flipped so the cover slip was placed at the bottom.

### Confocal microscopy of gut tissues

Fluorescently-labeled midgut tissues were imaged using a Leica SP8 inverted confocal microscope. For fluorescence excitation, the 405 nm fixed-wavelength laser was used for DAPI and a white light laser selective for wavelengths between 470 and 670 nm was used for tetramethylrhodamine and phalloidin. Images were taken with the following three wavelengths: red at Ex 542 nm, Em 572–759 nm for TMR-RK8 and TMR-Scrambled with a white light laser; blue at Ex 364, Em 410–480 for DAPI; and green at Ex 493, Em 498–555 for phalloidin. Tissues were imaged at two magnifications (20X and 40X objectives), optical sections were taken at steps of either 0.5, 1.1, or 2.9 μm along the Z-axis when using the 20X objective and at 0.5 μm steps when using the 40X objective. Lightning adaptive deconvolution, exclusive of Leica for this microscope model, was used to obtain maximum resolution of multiple images taken with the 40X objective. Images were analyzed and computer-colored using Fiji/ImageJ (National Institutes of Health, Bethesda, MD). Maximum intensity projections and videos of the z-stacks were then produced.

### Ligand competition assay

A ligand competition assay was conducted to verify the specificity of TMR-RK8 binding to the midgut^53^. Midguts were incubated in 1 µg/mL DAPI (200 µL) for 1 min and rinsed with 200 µL saline once. After saline removal, 200 µL (10 µM solution) of TMR-RK8 in tick saline was added to the tissue and incubated for 1 min before washing with tick saline solution twice, as before. The midgut was immediately imaged on the Leica SP8 inverted confocal microscope with the blue (for DAPI) and red (for TMR-RK8) settings (See *Confocal microscopy of gut tissues*). Following imaging, unlabeled RK8 kinin (200 µL of 100 µM) was added to the midgut, incubated for 1 min, and then imaged using the same setting to determine if the unlabeled RK8 displaced the TMR-RK8 kinin label.

### Tick midgut contraction assay

Midguts were dissected from immobilized female ticks under tick saline, as described under the *Midgut Dissection*. Ten different midguts were used for each of the two treatments, and ticks were of the same age and batch and had been maintained under the same conditions. Dissected midguts were placed individually into 200 µL of tick saline for 10 min at 28 °C on a gasketed glass coverslip (500 µL, Size 22 × 40 mm) serving as incubation chamber. After saline removal, the midgut was incubated in 200 µL of 10 µM of either TMR-Scrambled kinin or TMR-RK8 for 1 min and rinsed twice with 200 µL saline at 28ºC. After the final wash and removal of saline, 200 µL of saline were added, and a microscopy coverslip (24 × 60 mm, VWR, Radnor, PA) was placed over the gasketed portion of the incubation chamber, which height (0.5 mm) fits to prevent the midgut from floating, but still allows midgut peristalsis. The midguts were observed and filmed under a dissecting microscope (Olympus SZ61, Japan) equipped with an Infinity 5 camera (Teledyne Lumenera, Ottawa, ON, Canada). All videos were recorded for 1 min with the INFINITY ANALYZE 7 software (Teledyne Lumenera) at 30 frames/second with a 2464 × 2056 resolution. The field of view in all videos recorded represents 4 × 3.5 mm to capture the entire tick midguts under saline. All video files in Windows Media Video (.wmv) format obtained from the Infinity Analyze 7 software were converted to the MP4 format using CloudConvert (cloudconvert.com, Lunaweb, Munich, Germany) for analyses with the EthoVision XT 17 software (Noldus Information Technology, Wageningen, The Netherlands).

### Video analysis of midgut activity

We used the EthoVision 17 XT software to quantify the “activity” (pixel change) of the midguts from the videos recorded. This software defines activity as the percentage of pixels that have changed in the entire defined arena between the current image and previous image. The videos are recorded in 2464 × 2056 resolution and the arena is set to be the entire video screen. Video files were uploaded into the program and analyzed starting from time zero to the end of the 60 s video. Detection settings for activity included the activity threshold which was set to 5 to achieve zero detection when the midgut was immobile. Although the video captured 30 frames (images) per s, for the video analysis we chose a “sample rate” of 7 samples /s, with sample rate being the number of images from the video the EthoVision program analyzes per second. To calculate activity, each pixel of the video image was compared from one image to the next, and the proportion of pixel changes was calculated by dividing the number of pixels changed from one image to the next by the total number of pixels in the image. Care was given for the midgut to occupy most of the arena, as the activity analyses will reflect the midgut movement as a proportion of the total arena. Midgut activity is observed as pixel changes, which are represented by the red-colored pixels in the S4 video. The mean pixel change per midgut was then determined by averaging the pixel changes from all samples within the recording period. For each treatment, there were 10 biological replicates.

### Statistical analyses

All results were analyzed using GraphPad Prism v10.0.2 software (GraphPad Software Inc., San Diego, CA, USA). The dose–response curves of the two cell lines to the blank buffer, the TMR-labeled and unlabeled RK8 peptide at different concentrations were calculated with a simple linear regression with 4 replicates per treatment.

Comparisons of the midgut activity between the different treatments (10 replicates per treatment) were using a Mann–Whitney test. Results are presented as the mean ± standard deviation (SD). Statistical significance was defined as *P* < 0.05, with the *P* value displayed on each graph.

## Results

### Protein BLAST analysis of tick kinin receptors

An NCBI BLAST search using the translated *R. microplus* kinin receptor sequence (GenBank accession number: AAF72891.1) from Holmes, et al.^46^ as the query identified similar protein sequences from other tick species. Sequences annotated as ‘RYamide receptor isoform X1’ and ‘RYamide receptor isoform X2’ from *R. sanguineus* (LOC119395837) had 97.74% identity over 100% coverage and 97.92% identity over 84% coverage, respectively (Supplementary file S1). These, therefore, correspond to the kinin receptor from *R. sanguineus*. An alignment of these protein sequences revealed that most amino acid differences occur at the N-terminus, away from the orthosteric peptide kinin binding sites predicted in the hydrophobic pocket of the transmembrane regions^54,55^. A supplementary blastp analysis with the TSA database did not identify any more highly identical sequences (> 50%). These results support that the tick kinin receptors of both *R. microplus* and *R. sanguineus* are similar and are likely to have identical kinin binding sites. However, other RYamide predicted receptors of *R. sanguineus* are correctly annotated as such in NCBI; this is further shown in analyses and alignments presented in the Supplementary file S1.

### TMR-RK8 specifically activated the tick kinin receptor expressed in CHO-K1 cells

**Figure 1:**
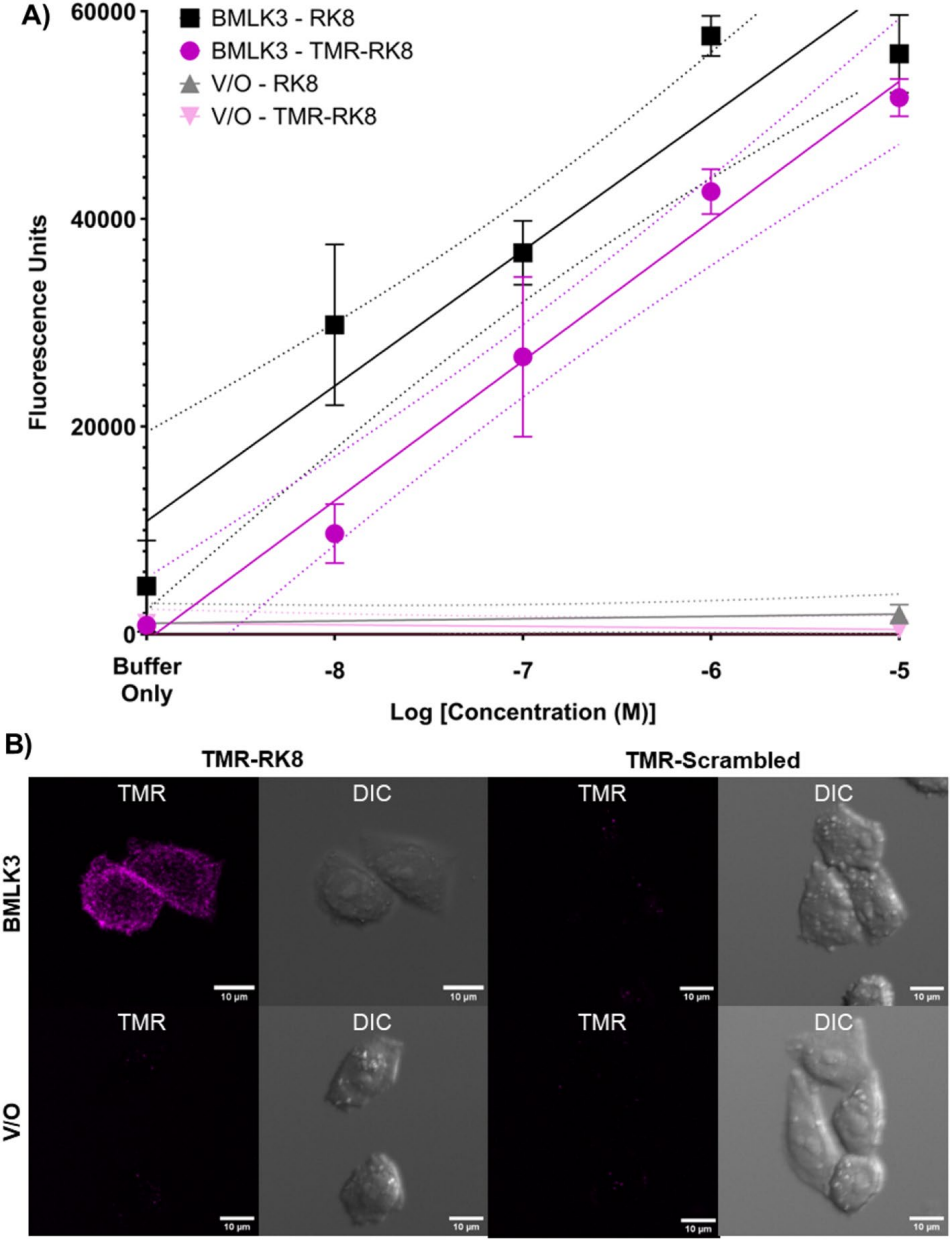
TMR-RK8 functionally activates the tick recombinant kinin receptor in BMLK3 cells. (**A**) The cell calcium fluorescence responses were recorded with Clariostar plate reader for 60 s with 1-s intervals after injection of different concentrations of the TMR-labeled or 10 µM of unlabeled RK8 peptide. Each column represents the average raw fluorescence units of 4 technical replicates of either the BMLK3 (black) or Vector-only (gray; V/O) cell lines’ response to the added peptide. Dose response curves were generated with a simple linear regression. V/O cells did not have a calcium fluorescence response at the highest concentration. Blank indicates application of buffer (1% DMSO in HBSS) only. (**B**) Live CHO-K1 cells expressing the tick kinin receptor (BMLK3) and vector-only (V/O) cell lines were treated with 10 µM of either TMR-RK8 or TMR-Scrambled prior to the capture of confocal images that show a maximum projection of confocal Z-stack series. TMR-RK8 signal (magenta) was observed only in BMLK3 cells. All images were taken using identical camera settings.

We validated the functional activity of TMR-RK8 on the recombinant CHO-K1 cell line BMLK3 developed by Holmes, et al.^47^ stably expressing the tick kinin receptor by utilizing a fluorescence-based calcium mobilization assay and fluorescence microscopy (Fig. 1). Ligand applications of either TMR-RK8 or unlabeled RK8 to the BMLK3 cells elicited a dose-dependent intracellular calcium fluorescence response, in contrast to the invariable background fluorescence of the vector only cells (Fig. 1A). Neither cell line showed fluorescence response when treated with only the blank buffer (1% DMSO in HBSS). The confidence intervals of the cell responses (fluorescence units) to TMR-RK8 and RK8 showed areas of overlap demonstrating the TMR fluorescent label did not impair peptide bioactivity, and the fluorescence units were maximal and similar for the highest concentration assayed of 10 µM (Fig. 1A). To further demonstrate the specificity and suitability of TMR-RK8 for receptor visualization, BLMK3 and the vector only cell lines were incubated with either TMR-RK8 or TMR-Scrambled and imaged. Only BMLK3 incubated with TMR-RK8 showed specific labeling, while BMLK3 incubated with TMR-Scrambled did not (Figs. 1B, S1). The vector only cells were not labeled by either peptide (Fig. 1B). The receptor was consistently visualized with TMR-RK8 across multiple BMLK3 cells (Figs. S1 and S2). Altogether these experiments validated TMR-RK8 specificity for the tick kinin receptor and its suitability for ex vivo assays.

### A fluorescent kinin 8 localized the kinin receptor in the tick midgut

**Figure 2:**
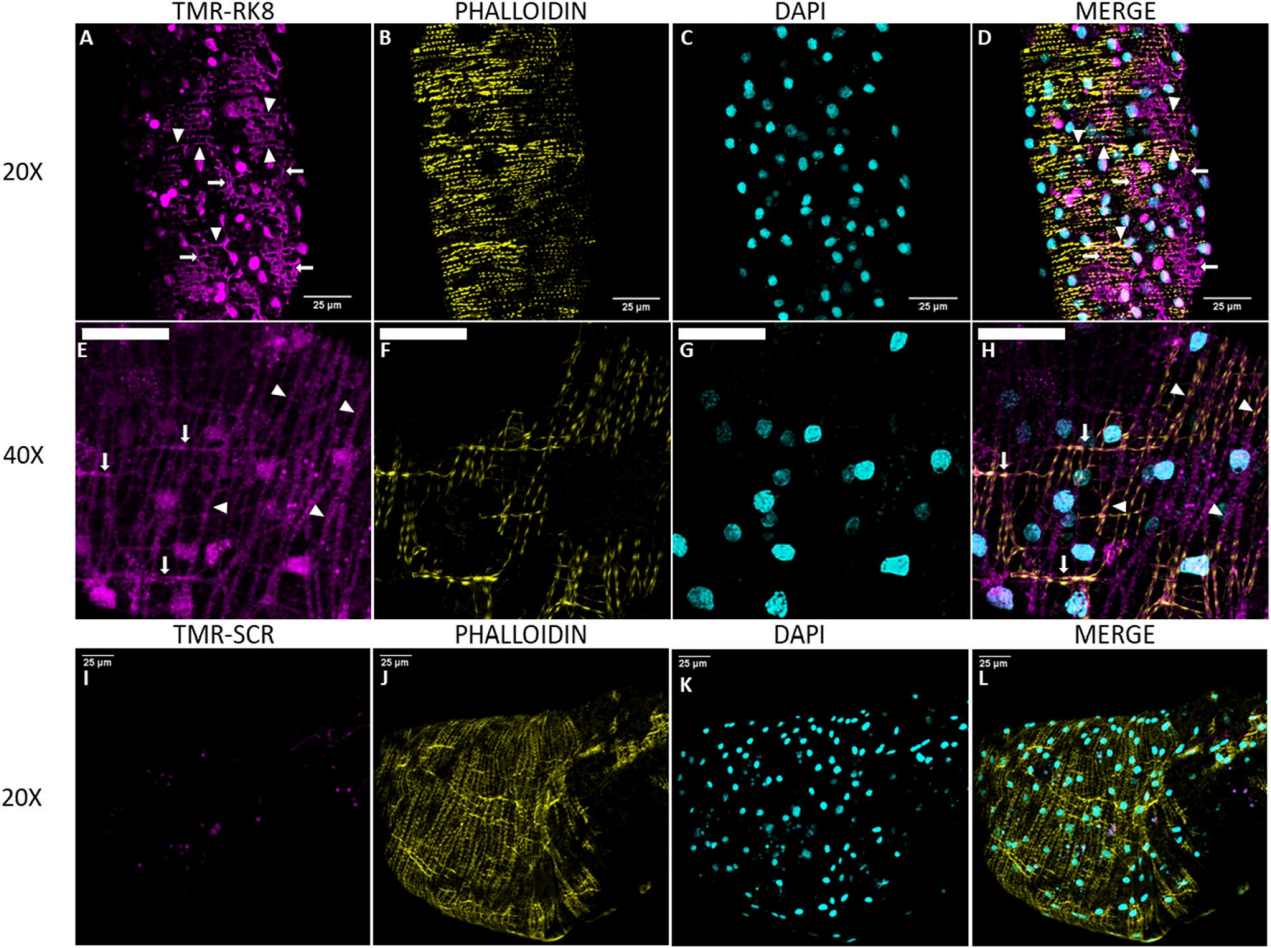
TMR-labeled tick kinin 8 (TMR-RK8) localized the kinin receptor on midgut peripheral muscles of *Rhipicephalus sanguineus*. Images show the maximum projection of confocal Z-stack series after application of either 10 µM solution of TMR-RK8 (**A**–**H**) or TMR-Scrambled (**I**–**L**; abbreviated as TMR-SCR here only) to midguts of individual females of *R. sanguineus*. (**A**–**H**) TMR-RK8 (magenta) signal was observed in the tick midgut muscles under confocal microscopy. In all panels, circular (white arrowheads) and longitudinal (white arrows) muscle fibers are visualized with TMR-RK8 (magenta). (**D**,**H**). The merged images show receptor signal overlapping, but clearly above the nuclei (magenta over cyan) and staining of TMR-RK8 interspaced with phalloidin signal in the muscles (magenta between yellow). (**I**–**L**) A labeled control kinin peptide of scrambled sequence (TMR-Scrambled, magenta) did not show specific binding to the midgut of ticks. Cell nuclei were stained with DAPI (cyan), and F-actin filaments were stained with Alexa 488-phalloidin (yellow). White scale bars indicate 25 µm. Images were acquired with a Leica SP8 inverted confocal microscope as Z-stacks using either a 20x/ 1.1 water immersion objective (**A**–**D**, **I**–**L**) or taken with lightning adaptive deconvolution using the 40X / 1.1 water immersion objective for maximal signal resolution (**E**–**H**). Z-stacks were taken as follows: (**A**–**D**), 0.5 µm Z-step, 37 slices; (**E**–**H**), 0.5 µm Z-step, 24 slices; (**I**–**L**), 2.9 µm Z-step, 35 slices.

In the midgut of *R. sanguineus*, application of TMR-RK8 localized the kinin receptor to both circular and longitudinal muscles, which were recognized by their typical mesh-like pattern (Fig. 2A). Furthermore, TMR-RK8 could be more clearly observed over the muscle fiber nuclei as the external plasma membrane was somewhat elevated by the nucleus volume in comparison to the rest of the fine muscle fiber (Fig. 2E, circular magenta signal). Employing lightning adaptive deconvolution allowed observation of the localization of the tick kinin receptor in longitudinal and circular muscles in the midgut periphery, distinct from the pattern of phalloidin binding to F actin (Fig. 2H). The TMR-Scrambled kinin peptide control did not significantly bind to the surface of the tick midgut, and tissues show no or minimal (Fig. 2I) TMR labeling (magenta), as expected (Fig. 2I–L).

In the S3 video the purple-labeled muscle fibers and the cytoplasm surrounding the muscle nuclei could be observed on the apical surface of the midgut (S3 Video). These results support that the binding observed from the labeled kinin was highly specific to the muscle fibers and prominently seen in the plasma membrane near muscle nuclei (see Fig. 2H, distinct cyan nuclei and plasma membrane magenta labeling, bottom left corner of the panel).

**Figure 3:**
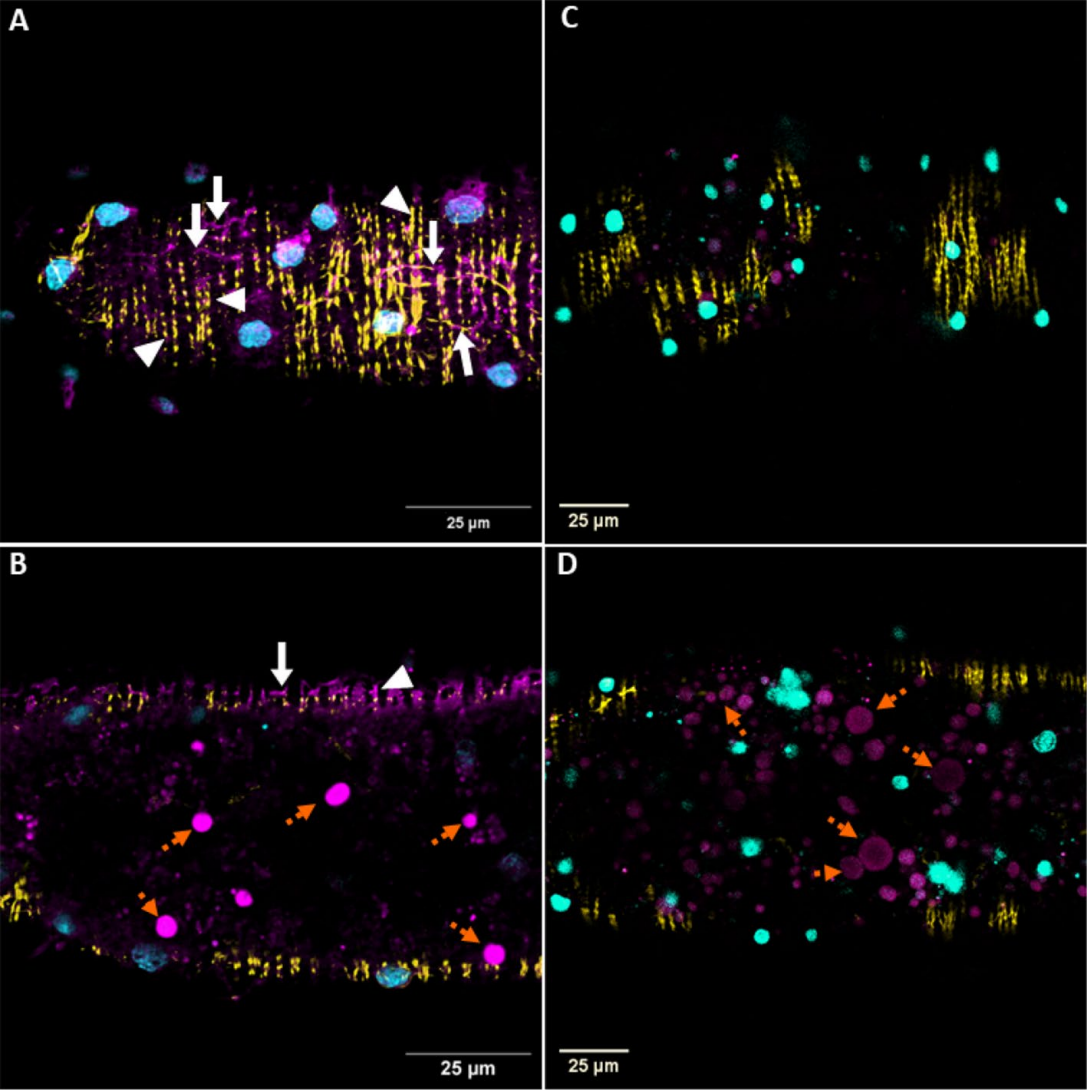
TMR-RK8 binds specifically to muscles on the surface of *Rhipicephalus sanguineus* midgut. Individual midguts of females *R. sanguineus* were treated with either 10 µM solution of TMR-RK8 or TMR-Scrambled (both magenta signals in **A**–**D**). Cell nuclei were stained with DAPI (cyan), and F-actin filaments were stained with Alexa 488-phalloidin (yellow). (**A**) Confocal Z-stack series of midgut surface after application of TMR-RK8 (magenta) showed the kinin receptor is localized on the surface of the tick midgut, binding specifically to the circular (white arrowheads) and longitudinal (white arrows) muscles; 0.5 µm Z-step, first 11 sections from the surface. (**B**) Z-slices under the surface of the midgut showed aggregation of TMR-RK8 signal in what appears to be lipid vesicles (orange dotted arrows)^56^ and muscles are labeled by TMR-RK8 on the midgut surface (white arrowheads); 0.5 µm Z-step, 19th section from the surface. (**C**) No signal of the TMR-Scrambled control kinin peptide was observed on the surface of the tick midgut; 2.9 µm Z-step, first 3 sections of the midgut surface. (**D**) TMR-Scrambled treatment also displayed non-specific aggregation in lipid vesicles beneath the midgut surface (orange dotted arrows); 2.9 µm Z-step, 7th section below the surface. White scale bars represent 25 µm. Images were acquired as Z-stacks using a 20x/ 1.1 water immersion objective.

Staining of the muscle fibers by TMR-RK8 was observed only on the surface of the midgut (Fig. 3A). The specific muscle surface staining was not observed when the midgut was treated with TMR-Scrambled (Fig. 3C). However, there was non-specific labeling for both the TMR-RK8 (Fig. 3B) and TMR-Scrambled treatments (Fig. 3D). This dotted pattern of non-specific labeling within the midgut was distinct from that of the surface musculature fibers and only occurred below the midgut surface in what appeared to be lipid vessicles^56^. Remarkably, localization of TMR-RK8 at the muscle fibers was observed even in the presence of phalloidin staining, indicating that the binding of TMR-RK8 to the tick kinin receptor was specific and did not compete for or overlap with the F actin binding site of phalloidin, which stains the interface between F-actin subunits, and there was no observed phalloidin displacement of the TMR-RK8 label (Fig. 3A).

**Figure 4:**
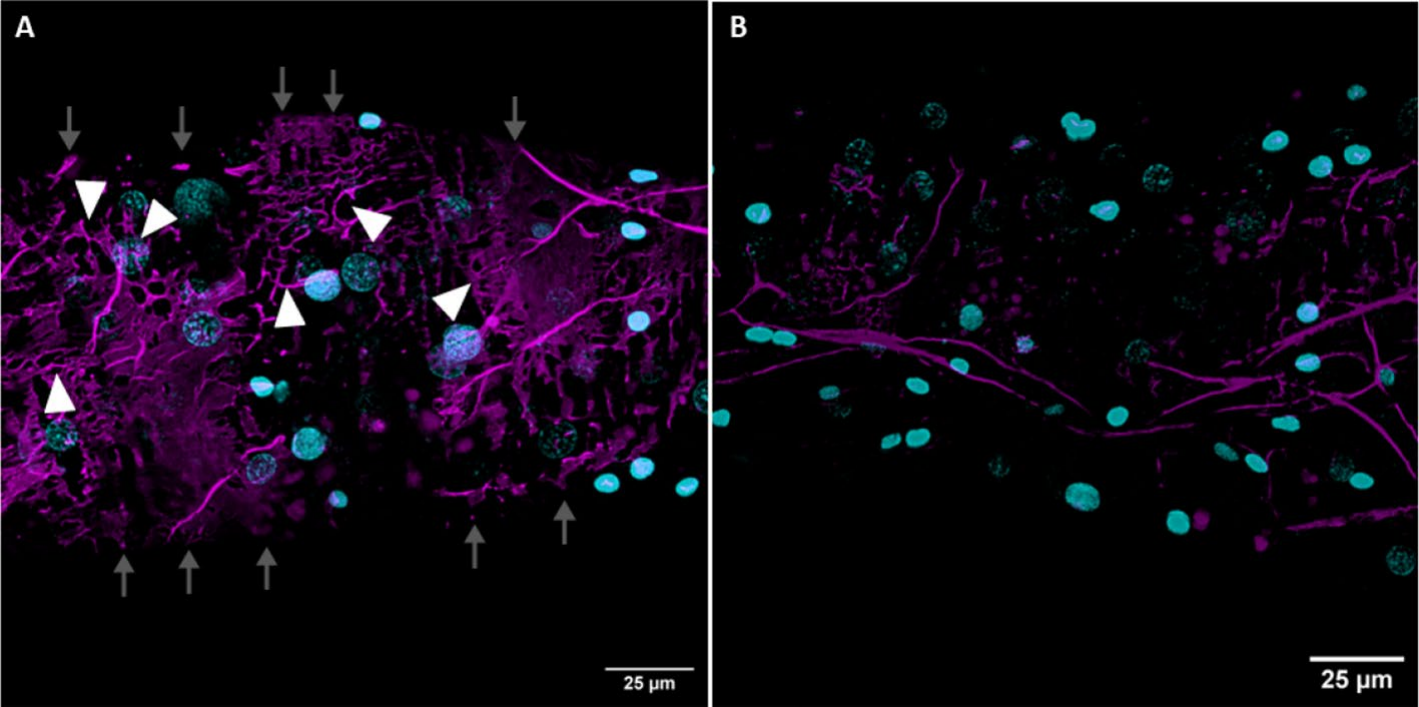
TMR-RK8 was competitively displaced by excess unlabeled RK8 peptide. (**A**) Application of TMR-RK8 (10 µM; magenta) to midguts localized tick kinin receptors on muscle fibers (white arrowheads) that were clearly observed in the midgut periphery (magenta, gray arrows); 1.1 µm Z-step, 35 sections. Nuclei were stained with DAPI. (**B**) Competition of TMR-RK8 (10 µM) with unlabeled RK8 (100 µM) greatly reduced the fluorescent signal of muscles, and the labelling of muscles disappeared in the midgut border so that the midgut contour definition was lost; 1.1 µm Z-step, 49 sections. White scale bars represent 25 µm. Images were acquired as Z-stacks using a 20x/ 1.1 water immersion objective.

Competition experiments with unlabeled RK8 led to a substantial reduction in the fluorescent signal observed on muscles compared to the muscle labeling by TMR-RK8 observed in the pre-competition image (Fig. 4A), indicating that the TMR-RK8 binding was effectively competed by unlabeled RK8 (Fig. 4B). These results provide strong evidence for the specific and selective binding of TMR-RK8 to tick kinin receptors on midgut muscles.

### The fluorophore-labeled kinin induces peristalsis of the tick midgut

**Figure 5:**
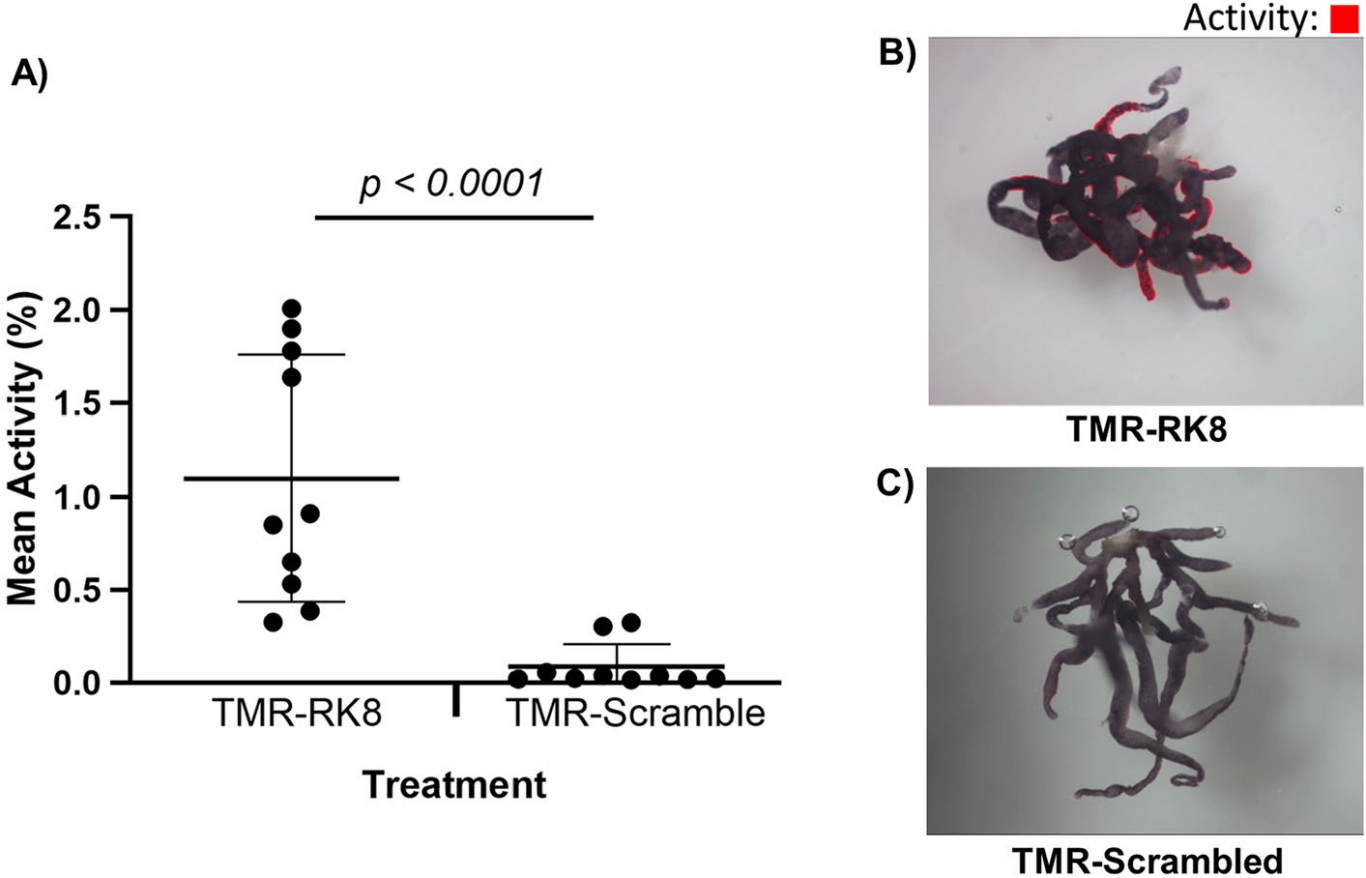
Application of TMR-RK8 induced contractions in midguts of *Rhipicephalus sanguineus* that were quantified by EthoVision analyses. (**A**) Mean activity (mean pixel change) obtained from the videos analyzed using EthoVision XT 17 (mean ± SD). A Mann–Whitney test was performed to determine the difference in activity between the two treatments on individual ticks; statistical significance is defined as *P* < 0.05. The *P* value is displayed above the histogram. (**B**) Videos analyzed by EthoVision demonstrated activity (in red) when the ex vivo midgut was treated with TMR-RK8, which induced peristalsis. (**C**) Midguts treated with the TMR-Scrambled peptide displayed low to imperceptible activity. Figures (**B**,**C**) are screenshots from Movies S4 and S5, respectively.

We investigated the effect of the labeled TMR-RK8 peptide on the midgut activity in comparison to the similarly labeled TMR-Scrambled peptide as a negative control by measuring the pixel changes recorded from midgut videos over time using the EthoVision software “activity analysis” function. In the video-tracking analysis, midguts exposed to TMR-RK8 (*N* = 10) had higher mean activity compared to those exposed to the TMR-Scrambled treatment (*N* = 10) for the duration of the videos (Fig. 5; Mann Whitney test, *P* = 0.011).

In the video recordings of the tick midguts contracting taken at 2464 × 2056 resolution, there were a total of 5,065,984 pixels in each “sample” (image) which were compared to the next. This analysis yielded a mean 1% activity from the TMR-RK8 treatment which in EthoVision means that there were about 50,660 pixels changing from each sample to the next. The videos of the TMR-Scrambled treatment resulted in less than 0.1% activity (i.e., there were an average of only 5066 pixels changing from each sample). Although 1% activity may appear to be low, it is important to note that the activity changes originated from the midgut contracting but the midgut occupied a small percentage of the arena. These activity changes were detected mainly from the edge of the midgut caeca contrasting against the white background and manifested either as clear peristaltic waves moving along the caeca or as lateral rhythmic movements or pulses of the caeca (Fig. 5B and Movie S4; areas where activity was detected are shown in red). Midguts exposed to the TMR-Scrambled did not display significant activity (Fig. 5C and Movie S5; only a flash of detected activity appears in red in the video). In sum, the TMR-RK8 treated midguts had more movement than the TMR-Scrambled treated midguts by a factor of 10. These results support that even with a fluorophore label, RK8 binding to the midgut induced the expected myotropic physiological response known for kinins in other arthropods, while the TMR-Scrambled peptide did not.

## Discussion

Given their significance in various insect physiological processes, deciphering the functions of kinins in ticks can offer valuable insights into tick feeding behavior, reproduction, and potential avenues for developing targeted interventions against tick-borne diseases. It was previously established that the tick kinin receptor transcript is expressed in the midgut of *Rhipicephalus microplus*^29^, which was recently confirmed by analyses of the midgut transcriptome (Rm gut CDS Nucl. Seq. Rm 24,078; 7tmA_leucokinin-like 1e−38), providing further confirmation of kinin receptor expression in the midgut^57^. Further, in our analyses of kinins from eight tick species the kinin 8 sequence cloned from *R. microplus* and the predicted from *R. sanguineus*^50^ were 100% identical. Kinin 8 was also highly active at the nanomolar level on the *R. microplus* recombinant receptor^52^ and was chosen for being one of the longest tick kinins (12 amino acid residues) to be N-labeled without potential label steric interference with the active kinin core at the C-terminus that binds the receptor. For the above reasons kinin 8 was selected for tick kinin receptor localization.

Visualizing GPCRs using fluorescent ligands is a valuable tool in modern pharmacology to characterize ligand-receptor interactions in targeted tissues^58^. In our investigation to localize the kinin receptor of the tick, we employed TMR-labeled specific and scrambled-sequence kinin peptides. The decision to use TMR-labeled peptides for receptor localization originated from the challenges encountered in generating anti-kinin receptor anti-peptide antibodies. Despite previous success in obtaining anti-kinin receptor antibodies in bovines which broadly immunolocalized the cattle tick kinin receptor in the midgut periphery of females of *Rhipicephalus microplus*^29^, our attempts in rabbits had failed to produce specific antibodies against it. To overcome this limitation and to obtain a more precise localization of the receptor, we now utilized a fluorophore-labeled peptide for localizing the tick kinin receptor, technique which had been successfully performed to localize the insect kinin receptor^53^. We previously determined that the minimal kinin core fragment retaining activity on the recombinant kinin *R. microplus* receptor consisted of the conserved C-terminal five amino acid residues characteristic of kinins, FX_1_X_2_WGa, where residues at X_1_ and X_2_ positions can be variable^59,60^. The TMR-RK8 activated the receptor in a dose response fashion, similarly to the unlabeled RK8 peptide (Fig. 1A). Thus, in this work, as expected, the labeling with tetramethyl rhodamine at the N-terminus of the relatively long, twelve-residue kinin 8 did not interfere with the ability of the C-terminus kinin core to either activate or label the receptor, as shown in the recombinant cell line expressing the tick kinin receptor (Fig. 1B, S1 and S2). These experiments unequivocally demonstrated both the specificity and activity of the TMR-RK8 to investigate the physiological function of kinins in tick tissues. The use of *R. sanguineus* to analyze the labeled kinin (TMR-RK8) activity on the ex-vivo midgut instead of *R. microplus* was obligated because of APHIS (USA) regulations that do not allow the shipping of live ticks outside the Cattle Fever Tick laboratory at the USDA-ARS in Edinburg, Texas, and the lack of a confocal microscope at that facility for imaging. An alignment of the amino acid sequences of the tick kinin receptor between the *R. sanguineus* and *R. microplus* demonstrated high similarity between the tick species (Supplementary File S1), with most of the few different amino acids at the N-terminus, away from the ligand binding pockets of most Class A GPCRs^61^ and specifically from the most similar mammalian neurokinin receptors^54^. As the kinin receptors of both tick species exhibited significant similarity, utilization of the *R. sanguineus* midgut was valid for functional ex vivo analyses.

The specific binding of TMR-RK8 in combination with confocal microscopy allowed the identification of the kinin receptor distribution in the periphery of the tick midgut, specifically in muscle fibers surrounding the caeca as a closed network (Fig. 2A–D)^45^. The precise localization of kinin receptors in midgut on the circular and longitudinal muscle fibers, clearly distinct from the location of actin on the same fibers, as revealed by labeling by the toxin phalloidin, is consistent with their role in regulating muscle activity (Fig. 2E–H) as known for the insect hindgut^62–64^. The tick kinin receptor localization in longitudinal (white arrows) and circular (white arrowheads) muscles is evident by the grid pattern revealed by the label (Fig. 2A,H) and it had not been previously observed in such detail when the kinin receptor was immunolabeled in *R. microplus* midgut, as only cross sections were analyzed by immunohistochemistry^29^. Furthermore, this grid-like fluorescent pattern was not observed in the midguts of ticks treated with the TMR-Scrambled (Fig. 2I–L). However, non-specific labeling observed in lipid vesicles inside the midgut^56^ for both TMR-RK8 and TMR-Scrambled highlights the usefulness of similarly labeled scrambled ligands as negative control (Fig. 3A–D). The competitive inhibition of fluorescent signal on muscle tissue in the presence of excess unlabeled RK8 (Fig. 4A,B) validates TMR-RK8 labeling as a specific, reliable probe for studying tick kinin receptor localization.

Our finding aligns with analogous results in insects, such as observations of kinin receptor labeling in circular muscles in the midgut of aphids with a fluorescently-labeled kinin, as in this work, and a grid-like pattern of kinin receptor expression in muscles in hindgut and rectal pads of *Drosophila* (Lkr-promoter driven-green-fluorescence protein expression)^65,66^. In insects, the kinin receptor was immunolocalized in and elicits myotropic activity of the hindgut^17,62,67^. The use of the EthoVision software to analyze contractions of the tick hindgut elicited by several neuropeptides had been previously reported^49^. Our results are the first to show the direct activity of a tick kinin neuropeptide on the tick midgut. The induction of midgut contractions by the labeled tick kinin 8 (Fig. 5) demonstrates both the specificity of the labeled ligand and the significance of kinin signaling in tick physiology. The functional consequences of TMR-RK8 binding, as evidenced by increased midgut contractions, may have important implications for our understanding of tick blood-feeding and its regulation. In dipterans, the kinin receptor in the stellate cells of Malpighian tubules regulates diuresis^17,68,69^. At the central nervous system level of Diptera, insect kinins are involved in meal size regulation^70,71^.

The expansion of kinins in some groups of arthropods such as ticks reveals the complexity for understanding this neuropeptide signaling. Various well-studied insects, including *Drosophila melanogaster*^72,73^, *Bombyx mori*^74^, and *Locusta migratoria*^75^ possess only one identified kinin peptide, while others such as *Rhodnius prolixus* and *Leucophaea maderae* possess multiple kinins^67,76^. Additionally, there are insects in which no kinin has been observed such as in the model coleopteran *Tribolium castaneum*^77^ and in ants^78^. However, in *Pogonus chalceus*, a non-Polyphaga coleopteran, the insect kinin has not been lost ^79^. The number of kinin paracopies in hard ticks includes 11 in *Dermacentor variabilis*, 15 in *Amblyomma sculptum*, 16 in *Ixodes ricinus* and *Hyalomma dromedarii*, 17 in *R. sanguineus* and *R. microplus,* and 19 in *Ixodes scapularis*^50^. This expansion of kinins in the same transcript in ticks could represent an efficient way for obtaining high kinin peptide concentrations in hemolymph at specific times. Collectively, these findings contribute to the understanding of myotropic peptide receptors, particularly kinin receptors, across diverse arthropod species, offering insights into their evolutionary conservation and functional adaptations in relation to feeding and digestive processes^64^.

Previously, we demonstrated that silencing the kinin receptor of *R. microplus* has a fitness cost by decreasing the weights of egg masses and the number of eggs hatched per egg mass, as well as delaying the pre-oviposition and incubation periods^29^. Taken together our findings suggest that the kinin receptor-mediated midgut muscle contractions may play a role in facilitating movement of the acquired blood and its digestion within the tick midgut.

## Conclusion

We demonstrated the specific localization and functional relevance of the tick kinin receptor in the midgut of non-blood fed *R. sanguineus* tick females using a fluorophore-labeled kinin. These findings provide a foundation for future research in understanding the role of kinin signaling in tick physiology. More broadly, this technique may allow localization of other peptide GPCRs eliminating the need for anti-receptor antibody development and validation. Further investigations into the specific signaling mechanisms and downstream effects of kinin receptor activation in the midgut are warranted to fully comprehend their role in tick blood-feeding and in pathogen-midgut interactions. Antagonists of the kinin receptor may offer promise to disrupt tick midgut movement and digestion.

### Supplementary Information

Supplementary Video S3.

Supplementary Video S4.

Supplementary Video S5.

Supplementary Figure S1.

Supplementary Figure S2.

Supplementary Information.

## Data Availability

All data generated or analyzed during this study are included in this published article (and its Supplementary Information files).
