# Supplementary figures and images for: A fluorescently-tagged tick kinin neuropeptide triggers peristalsis and labels tick midgut muscles
Source: Sci Rep. 2024 May 13;14:10863. doi: 10.1038/s41598-024-61570-w (PMC11091117; doi:10.1038/s41598-024-61570-w)

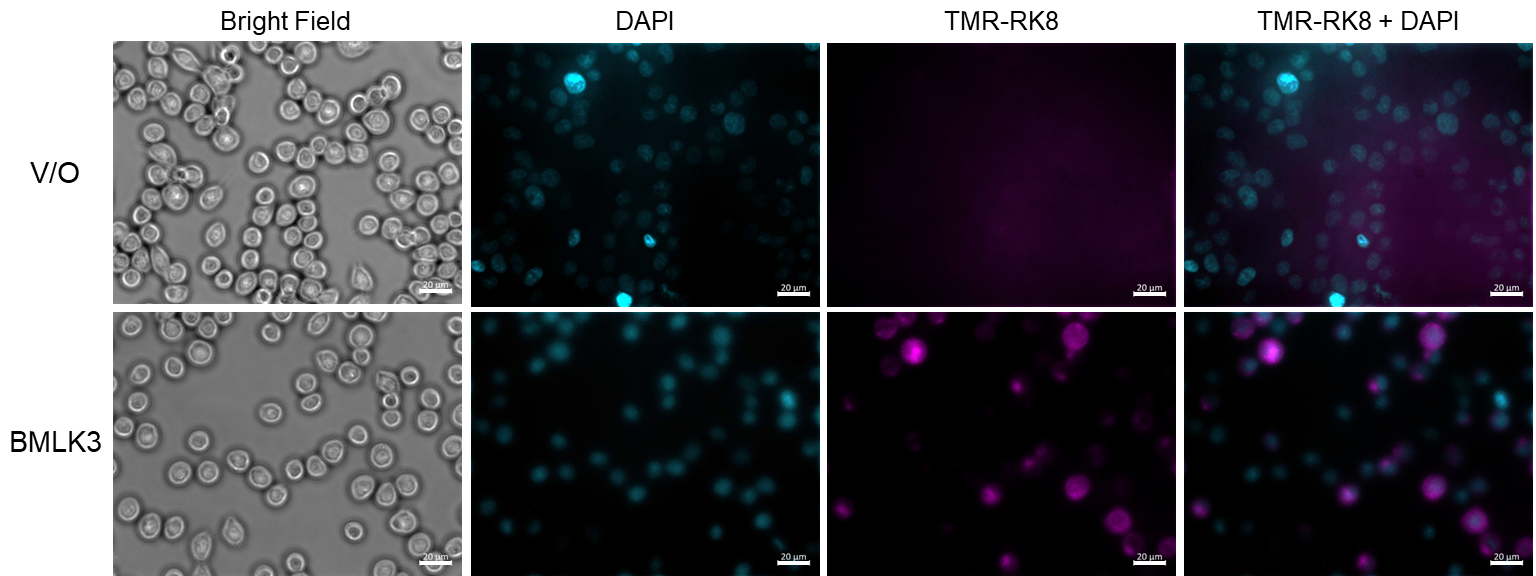

Supplement: Supplementary file 4 — Supplementary Figure S1. [file 41598_2024_61570_MOESM4_ESM.tif]

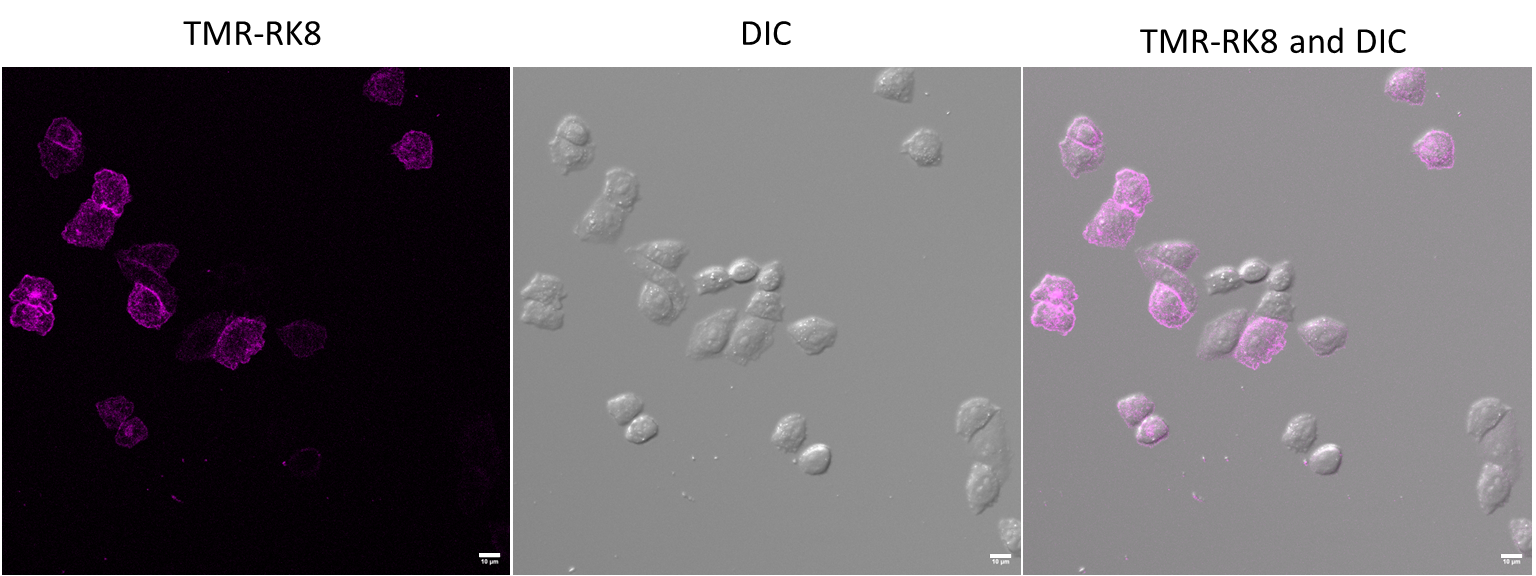

Supplement: Supplementary file 5 — Supplementary Figure S2. [file 41598_2024_61570_MOESM5_ESM.tif]
