# Supplementary material for: A fluorescently-tagged tick kinin neuropeptide triggers peristalsis and labels tick midgut muscles
Source: Sci Rep. 2024 May 13;14:10863. doi: 10.1038/s41598-024-61570-w (PMC11091117; doi:10.1038/s41598-024-61570-w)
Supplement: Supplementary file 6 — Supplementary Information. [file 41598_2024_61570_MOESM6_ESM.docx]

**SI Methods**

***Validation of the specificity of the fluorescently-labeled peptide on live cells***

BMLK3 and vector-only cells were cultured in selective media (F-12K medium containing 10% fetal bovine serum and 800 µg/mL-1 of G418 sulfate), cells were seeded in 6-well plates (CELLSTAR®, Greiner Bio-One®, Monroe, NC USA) at 20,000 cells per well and were incubated overnight. The next assay, the cell culture medium was removed, and cells were rinsed twice with 200 µl DPBS, and 180 µl of HBSS buffer was added into each well. The peptide stock solutions were prepared in 1% DMSO in HBSS at 100 µM. TMR-RK8 (20 µl) was added into each well and incubated with the cells for 5 min at room temperature. The medium was then removed with a pipette and the cells were rinsed twice with 200 µl HBSS, and DAPI (VECTASHIELD® H-1200, Vector Laboratories) was added (20 µl of 0.15 µg/ml) for visualizing the cell nuclei. Cells were rinsed twice with 200 µl of HBSS, and finally 180 µl HBSS per well was added.

The cells were immediately imaged under a Zeiss Axio Observer® inverted fluorescence microscope. The exposure time for the Bodipy-Tetramethylrhodamine-X (BPTmX) fluorescence channel was the same for BMLK3 cells and V/O cells. DAPI imaging was done subsequently using the same focal height as the BPTmx channel.

**SI Results**

***Tick kinin receptor alignment***

The functionally validated sequence of the leucokinin receptor protein from *R. microplus* AAF72891.1 was used in a blastp search against Acari and *Rhipicephalus sanguineus* databases in NCBI. The R. microplus receptor protein identified the predicted sequence with the annotation *Rhipicephalus sanguineus* RYamide receptor (LOC119395837), transcript variant X1, mRNA. Sequence ID: [XM_037662848.2](https://www.ncbi.nlm.nih.gov/nucleotide/XM_037662848.2?report=genbank&log$=nuclalign&blast_rank=1&RID=1DE7XVCC016)Length: 1365; protein_id=""[XP_037518776.1](https://www.ncbi.nlm.nih.gov/protein/1932651206)", which indeed corresponds to the tick leucokinin receptor. This RYa designation is incorrect because the alignment below clearly shows that is the orthologous *R. sanguineus* leucokinin receptor.

Accession | Description | [Species name] | Amino acid #

AAF72891.1 Leucokinin-like peptide receptor [*Rhipicephalus microplus*] 397

[XP_037518776.1](https://www.ncbi.nlm.nih.gov/protein/XP_037518776.1?report=genbank&log$=protalign&blast_rank=1&RID=0) Ryamide receptor isoform X1 [*Rhipicephalus sanguineus*] 398

Annotations

Yellow highlight – Amino acid replacement

**Bold and Underlined** – Amino acids corresponding to predicted transmembrane region, as annotated by Holmes, et al. ^1^.

RYamide receptor isoform X1 [*Rhipicephalus sanguineus*]

Max Score: 798; Total Score: 798; Query Cover 100%

E value: 0.0; Identities (%): 389/398 (97.74%); Positives (%): 392/398(98%)

Gaps (%): 1/398(0%)

Accession: **XP_037518776.1**

TM1

AAF72891.1 1 MTSLPGMTLDPSAPPPLLLDSSYVSPDYGNLSLLSSLPAANISSNKLYQV**PVGFIVLLSI** 60

MTSLPGMT DPSAPPPLLLDS Y SPDYGNLSLL SLPAAN+SS KLYQVPVGFIVLLSI

XP_037518776.1 1 MTSLPGMTHDPSAPPPLLLDSPYASPDYGNLSLLPSLPAANVSSGKLYQVPVGFIVLLSI 60

TM1 TM2

AAF72891.1 61 **FYGIISLVAVAGNFMVM**WIVATSR**RMQTVTNFFIANLAVADIIIGLFSIP**FQFQAALLQR 120

FYGIISLVAVAGNFMVMWIVATSRRMQTVTNFFIANLAVADIIIGLFSIPFQFQAALLQR

XP_037518776.1 61 FYGIISLVAVAGNFMVMWIVATSRRMQTVTNFFIANLAVADIIIGLFSIPFQFQAALLQR 120

TM3 TM4

AAF72891.1 121 WVLPEFMCA**FCPFVQVLSVNVSIFTLTAIAL**DRYRAVMSPLKARTTKLRAK**FIICGIWTL** 180

WVLPEFMCAFCPFVQVLSVNVSIFTLTAIALDRYRAVMSPLKARTTKLRAKFIICGIWTL

XP_037518776.1 121 WVLPEFMCAFCPFVQVLSVNVSIFTLTAIALDRYRAVMSPLKARTTKLRAKFIICGIWTL 180

TM4 TM5

AAF72891.1 181 **AVAAALPCALA**LRVETQVESHALNLTKPFCHEVGISRK**AWRIYNHVLVCLQYFFPLLTIC** 240

AVAAALPCALALRVETQVESHALNLTKPFCHEVGISRKAWRIYNHVLVCLQYFFPLLTIC

XP_037518776.1 181 AVAAALPCALALRVETQVESHALNLTKPFCHEVGISRKAWRIYNHVLVCLQYFFPLLTIC 240

TM5 TM6

AAF72891.1 241 **F**VYARMGLKLKESKSPGNAQGARDAGILKNKKKVIK**MLFVIVALFAFCWLPYQLYNILR**E 300

FVYARMGLKLKESKSPGNAQGARDAGILKNKKKVIKMLFVIVALFAFCWLPYQLYN+LRE

XP_037518776.1 241 FVYARMGLKLKESKSPGNAQGARDAGILKNKKKVIKMLFVIVALFAFCWLPYQLYNVLRE 300

TM7

AAF72891.1 301 VFPKIDKYKYIN**IIWFCTHWLAMSNSCYNPFIYAIY**NERFKREFATRCTCGGHRYKSPKS 360

VFPKIDKYKYINIIWFCTHWLAMSNSCYNPFIYAIYNERFKREFATRCTCGGHRYKSPKS

XP_037518776.1 301 VFPKIDKYKYINIIWFCTHWLAMSNSCYNPFIYAIYNERFKREFATRCTCGGHRYKSPKS 360

AAF72891.1 361 RFASYEQED-NSTIIVSMRHSFRLSFKNSAPLKASTQV 397

RFASYEQED NSTIIVSMRHSFRLSFKNS+PLKASTQV

XP_037518776.1 361 RFASYEQEDNNSTIIVSMRHSFRLSFKNSSPLKASTQV 398

Sequence alignment of the leucokinin receptor from *R. microplus* with annotated RYa receptors from *R. sanguineus* showing that the sequence XP_037518776.1 is incorrectly annotated and indeed correspond to the leucokinin receptor. The shorter sequence towards the C-terminal end is correctly predicted as putative leucokinin-like receptor of *R. sanguineus.*

**AAF72891.1**

leucokinin-like peptide receptor [*Rhipicephalus microplus*]

**ACX33154.1** Length: 83. (81/83; 98%); positives (82/83; 98%) and gaps (1/83; 1%).

putative leucokinin-like receptor [*Rhipicephalus sanguineus*]

**XP_037518776.1** Length: 398. Identities (389/398; 98%); positives (392/398; 98%) and gaps (1/398; 0%).

RYamide receptor isoform X1 [*Rhipicephalus sanguineus*]

AAF72891.1 1 MTSLPGMTLDPSAPPPLLLDSSYVSPDYGNLSLLSSLPAANISSNKLYQVPVGFIVLLSIFYGIISLVAVAGNFMVMWIV 80

ACX33154.1 --------------------------------------------------------------------------------

XP_037518776.1 1 MTSLPGMTHDPSAPPPLLLDSPYASPDYGNLSLLPSLPAANVSSGKLYQVPVGFIVLLSIFYGIISLVAVAGNFMVMWIV 80

AAF72891.1 81 ATSRRMQTVTNFFIANLAVADIIIGLFSIPFQFQAALLQRWVLPEFMCAFCPFVQVLSVNVSIFTLTAIALDRYRAVMSP 160

ACX33154.1 --------------------------------------------------------------------------------

XP_037518776.1 81 ATSRRMQTVTNFFIANLAVADIIIGLFSIPFQFQAALLQRWVLPEFMCAFCPFVQVLSVNVSIFTLTAIALDRYRAVMSP 160

AAF72891.1 161 LKARTTKLRAKFIICGIWTLAVAAALPCALALRVETQVESHALNLTKPFCHEVGISRKAWRIYNHVLVCLQYFFPLLTIC 240

ACX33154.1 --------------------------------------------------------------------------------

XP_037518776.1 161 LKARTTKLRAKFIICGIWTLAVAAALPCALALRVETQVESHALNLTKPFCHEVGISRKAWRIYNHVLVCLQYFFPLLTIC 240

AAF72891.1 241 FVYARMGLKLKESKSPGNAQGARDAGILKNKKKVIKMLFVIVALFAFCWLPYQLYNILREVFPKIDKYKYINIIWFCTHW 320

ACX33154.1 1 ---------------------------------------------------------------------------FCTHW 5

XP_037518776.1 241 FVYARMGLKLKESKSPGNAQGARDAGILKNKKKVIKMLFVIVALFAFCWLPYQLYNVLREVFPKIDKYKYINIIWFCTHW 320

AAF72891.1 321 LAMSNSCYNPFIYAIYNERFKREFATRCTCGGHRYKSPKSRFASYEQED-NSTIIVSMRHSFRLSFKNSAPLKASTQV 397

ACX33154.1 6 LAMSNSCYNPFIYAIYNERFKREFATRCTCGGHRYKSPKSRFASYEQEDNNSTIIVSMRHSFRLSFKNSSPLKASTQV 83

XP_037518776.1 321 LAMSNSCYNPFIYAIYNERFKREFATRCTCGGHRYKSPKSRFASYEQEDNNSTIIVSMRHSFRLSFKNSSPLKASTQV 398

*I.sc.* [XP_040075533.1](https://www.ncbi.nlm.nih.gov/protein/XP_040075533.1?report=genbank&log$=protalign&blast_rank=0&RID=1BMB0T56013) 1 MD-----S-TNGPSAPPTATSNWTSQPASTESAACDLPPPVPEGMQALMYIMYIAVSVAAIGGNGIVCYIVLAYQRMRTV 74

*R.m.* [XP_037270636.1](https://www.ncbi.nlm.nih.gov/protein/XP_037270636.1?report=genbank&log$=protalign&blast_rank=1&RID=1BMB0T56013) 1 METESELSLSSGEEASPSAASNWSGGNGLDAAASCDVSPQVPEGIQALMYLMYIAVSVAAIGRNGIVCYIVIAYQRMRTV 80

*R.s.* [XP_037504890.1](https://www.ncbi.nlm.nih.gov/protein/XP_037504890.1?report=genbank&log$=protalign&blast_rank=2&RID=1BMB0T56013) 1 METESELS-SSVEEASPSAATNWSGGNGSEAAASCDVSPQVPEGIQALMYLMYIAVSVAAIGGNGIVCYIVIAYQRMRTV 79

[XP_040075533.1](https://www.ncbi.nlm.nih.gov/protein/XP_040075533.1?report=genbank&log$=protalign&blast_rank=0&RID=1BMB0T56013) 75 TNMFIMNLAIGDILMASLCIPFTFVSNLLLGYWPFGGVMCVVVTYAQCVTVFISAYTLIAISVDRYTAIVYPLRPRMTKL 154

[XP_037270636.1](https://www.ncbi.nlm.nih.gov/protein/XP_037270636.1?report=genbank&log$=protalign&blast_rank=1&RID=1BMB0T56013) 81 TNMFIMNLAIGDILMACLCIPFTFVSNLLLGYWPFGGVMCVLVTYAQCVTVFISAYTLIAISVDRYTAIVYPLRPRMSKL 160

[XP_037504890.1](https://www.ncbi.nlm.nih.gov/protein/XP_037504890.1?report=genbank&log$=protalign&blast_rank=2&RID=1BMB0T56013) 80 TNMFIMNLAIGDILMACLCIPFTFVSNLLLGYWPFGGVMCVLVTYAQCVTVFISAYTLIAISVDRYTAIVYPLRPRMTKL 159

[XP_040075533.1](https://www.ncbi.nlm.nih.gov/protein/XP_040075533.1?report=genbank&log$=protalign&blast_rank=0&RID=1BMB0T56013) 155 RSKIIIGVVWLVALVTPLPTALVTQLVPHPCANQTYYCLEQWGTPEQTTYYSMALMILQYFFPLLALIFTYTRIAVVVWG 234

[XP_037270636.1](https://www.ncbi.nlm.nih.gov/protein/XP_037270636.1?report=genbank&log$=protalign&blast_rank=1&RID=1BMB0T56013) 161 RSKLIIALVWLVALVTPLPTALVTQLVPHPCANRTYYCLEQWGRPEQTAYYSMALMILQYFFPLLVLIFTYTRIAVVVWG 240

[XP_037504890.1](https://www.ncbi.nlm.nih.gov/protein/XP_037504890.1?report=genbank&log$=protalign&blast_rank=2&RID=1BMB0T56013) 160 RSKLIIALVWLVALVTPLPTALVTQLVPHPCANRTYYCLEQWGRPEQTAYYSMALMILQYFFPLLVLIFTYTRIAVVVWG 239

[XP_040075533.1](https://www.ncbi.nlm.nih.gov/protein/XP_040075533.1?report=genbank&log$=protalign&blast_rank=0&RID=1BMB0T56013) 235 KETPGEAQDERDQRMAASKRKMIKMMIACVAAFLLCWLPLNLFIVVSEQYPDVYDLNGIGYVWFVCHWLAMSHTCYNPLI 314

[XP_037270636.1](https://www.ncbi.nlm.nih.gov/protein/XP_037270636.1?report=genbank&log$=protalign&blast_rank=1&RID=1BMB0T56013) 241 KETPGEAQDARDQRMAASKRKMTKMMITVVTVFMLSWLPLNTYILLSDLDPGVNNYEHIRYVYFIIHRLAMSHASYNPLI 320

[XP_037504890.1](https://www.ncbi.nlm.nih.gov/protein/XP_037504890.1?report=genbank&log$=protalign&blast_rank=2&RID=1BMB0T56013) 240 KETPGEAQDARDQRMAASKRKV---------------------------------------------------------- 261

[XP_040075533.1](https://www.ncbi.nlm.nih.gov/protein/XP_040075533.1?report=genbank&log$=protalign&blast_rank=0&RID=1BMB0T56013) 315 YFWMNAKFRTGLQAVFR----CW-------HVPKKKSSCFVSTVKK---------VSSASGNTT---------------- 358

[XP_037270636.1](https://www.ncbi.nlm.nih.gov/protein/XP_037270636.1?report=genbank&log$=protalign&blast_rank=1&RID=1BMB0T56013) 321 YCWMNAKFRDRFCQLFRRSKLCWPSRLRHQRPLRKESAAEVAALRRCNTYTTYVSVRAVPGSSYRFTKDAAQTNGKPKRY 400

[XP_037504890.1](https://www.ncbi.nlm.nih.gov/protein/XP_037504890.1?report=genbank&log$=protalign&blast_rank=2&RID=1BMB0T56013) --------------------------------------------------------------------------------

[XP_040075533.1](https://www.ncbi.nlm.nih.gov/protein/XP_040075533.1?report=genbank&log$=protalign&blast_rank=0&RID=1BMB0T56013) -----

[XP_037270636.1](https://www.ncbi.nlm.nih.gov/protein/XP_037270636.1?report=genbank&log$=protalign&blast_rank=1&RID=1BMB0T56013) 401 EDSRV 405

[XP_037504890.1](https://www.ncbi.nlm.nih.gov/protein/XP_037504890.1?report=genbank&log$=protalign&blast_rank=2&RID=1BMB0T56013) -----

Alignment of predicted RYamide receptors from, *Ixodes scapularis* [XP_040075533.1](https://www.ncbi.nlm.nih.gov/protein/XP_040075533.1?report=genbank&log$=protalign&blast_rank=0&RID=1BMB0T56013), *Rhipicephalus microplus* [XP_037270636.1](https://www.ncbi.nlm.nih.gov/protein/XP_037270636.1?report=genbank&log$=protalign&blast_rank=1&RID=1BMB0T56013)*,* and *Rhipicephalus sanguineus* [XP_037504890.1](https://www.ncbi.nlm.nih.gov/protein/XP_037504890.1?report=genbank&log$=protalign&blast_rank=2&RID=1BMB0T56013). The *Drosophila melanogaster* RYamide receptor protein P25931.2 was used as query for a tblastn search against the Ixodidae database in NCBI and identified the transcripts (XM_040219599.1; *Ixodes scapularis* RYamide receptor (LOC8032994), transcript variant X2, mRNA; XM_037414739.1; *Rhipicephalus microplus* RYamide receptor-like (LOC119162284), mRNA; XM_037648962.2) for the three above aligned receptor proteins.

Further analysis by aligning the *R. microplus* leucokinin receptor with the correctly annotated RYamide receptor from *R. sanguineus* clearly shows that they are not orthologous receptors, as shown below.

R.m. 49 QVPVGFIVLLSIFYGIISLVAVAGNFMVMWIVATSRRMQTVTNFFIANLAVADIIIGLFS 108

R.s. 39 ...E.IQA.MYLM.IAV.VA.IG..GI.CY..IAYQ..R....M..M...IG..LMACLC 98

R.m. 109 IPFQFQA-ALLQRWVLPEFMCAFCPFVQVLSVNVSIFTLTAIALDRYRAVMSPLKARTTK 167

R.s. 99 ...T.VSNL..GY.PFGGV..VLVTYA.CVT.FI.AY..I..SV...T.IVY..RP.M.. 158

R.m 168 LRAKFIICGIWTLAVAAALPCALALRVETQVESHALNLTKPFCHEVGISRKAWRIYNHVL 227

R.s. 159 ..S.L..ALV.LV.LVTP..T..V----..LVP.PCANRTYY.L.QWGRPEQTAY.SMA. 214

R.m 228 VCLQYFFPLLTICFVYARMGLKLKESKSPGNAQGARDAGILKNKKKV 274

R.s. 215 MI........VLI.T.T.IAVVVWGKET..E..D...QRMAAS.R.. 261

Sequence alignment of the leucokinin-like peptide receptor [*Rhipicephalus microplus* R.m.] ID: AAF72891.1, Length: 397 with the *R. sanguineus* (R.s.) RYamide receptor-like ID: [XP_037504890.1](https://www.ncbi.nlm.nih.gov/protein/XP_037504890.1?report=genbank&log$=protalign&blast_rank=1&RID=1BR8TE85114), Length: 261 (this sequence is also shown in the alignment above with other correctly predicted Ixodidae RYa receptors).

In this alignment: identities (90/227; 40%); positives (131/227; 57%) and gaps (5/227; 2%). Identities are indicated by dots.

***Supplementary Figures***


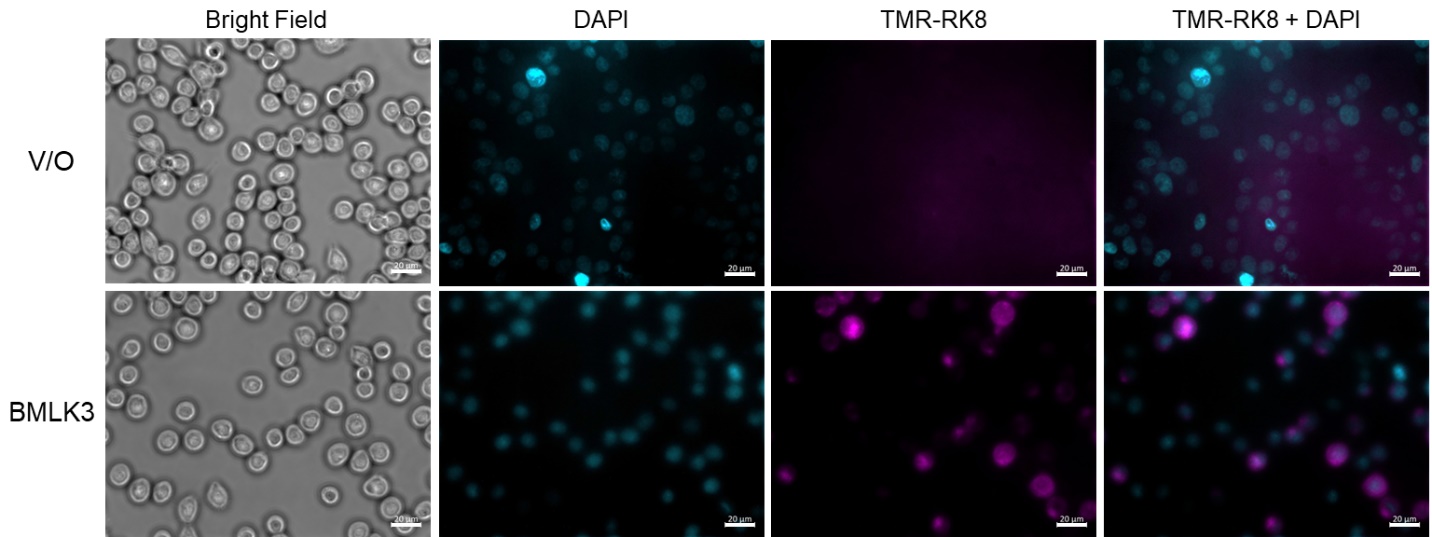


Figure S1. Fluorescence microscopy of multiple live CHO-K1 cells expressing the tick kinin receptor (BMLK3) or the Vector-only (V/O) cells treated with 1 µM of TMR-RK8. For each cell line, the left panel shows the bright field image of the corresponding three panels to the right. The DAPI panel only shows the nuclei of cells. The third panel shows that the TMR-RK8 signal (magenta; BPTmX fluorescence channel) was only observed in the BLMK3 cells. The TMR-RK8 labelling is not uniformly shown in all cells due to their different focal points when growing on the plate. The last panel shows merget fluorescent signals. All images were taken using identical camera settings with an Axio Observer inverted fluorescence microscope. The scale shown indicates 20 µm.


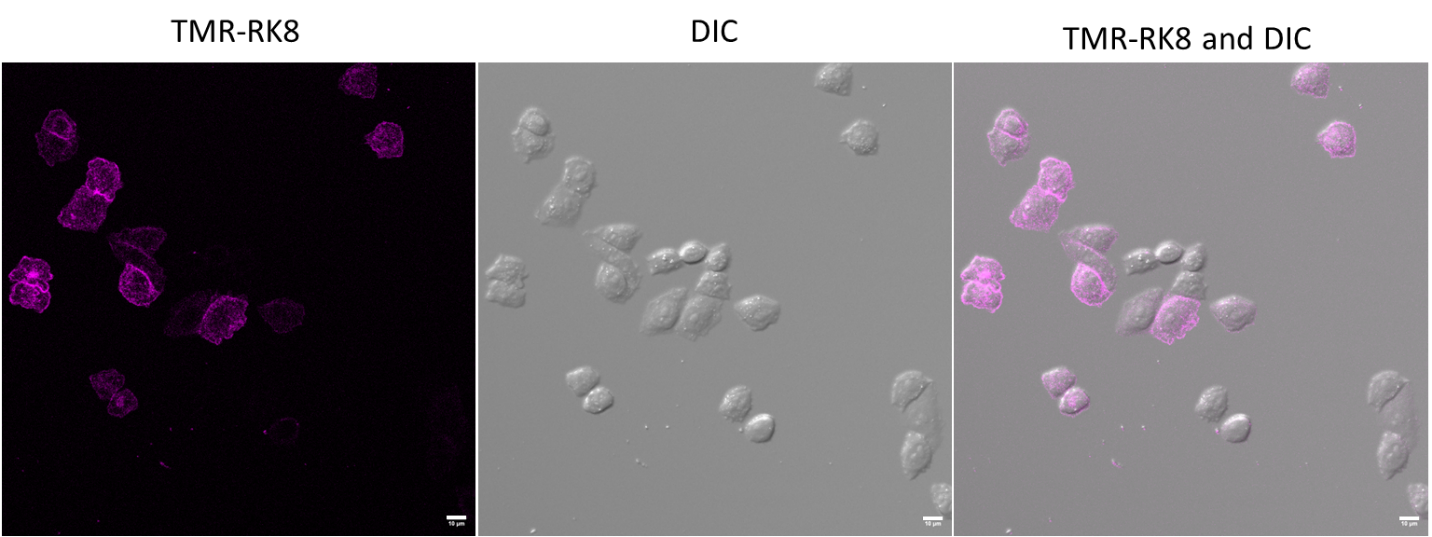


Figure S2. Confocal fluorescence microscopy combined with DIC (Nomarski) of the same live BMLK3 cells expressing the tick kinin receptor treated with 1 µM of TMR-RK8. Images show the maximum projection of the confocal Z-stack series after application of 1 µM of TMR-RK8. TMR-RK8 signal (magenta) was observed in most cells (left and right panels). The Z-stack was taken as follows: 1.1 µm Z-step, 13 slices with a Leica SP8 microscope. The scale shown indicates 10 µm.

S3 Video. Video recording of a Z-stack showing localized kinin receptors (magenta) on muscle cells on the midgut periphery of *Rhipicephalus sanguineus.* This video was taken with lightning adaptive deconvolution with the 40X objective and is the file from which Figures 2A and B were produced. To create the movie, the DAPI (cyan), phalloidin (yellow), and TMR-RK8 (magenta) signals were captured for each optical slice of the Z-stack (20.5 µm; 0.5 µm/section, 41 optical sections

S4 Video. Video of tick midgut treated with 1 µM TMR-RK8 showing induced peristalsis. Video recording of tick midgut contraction after 1 min of exposure at 4X speed. The video was analyzed in EthoVision XT 17 with activity analysis showing changes in activity (red). Rapid contractions were observed shortly after treatment with TMR-RK8.

S5 Video. Video of tick midgut treated with 1 µM of TMR-Scrambled showing minimal movement. Video recording of tick midgut after 10 min of exposure at 4X speed. The video was loaded in EthoVision XT 17 with activity analysis showing changes in activity (red). Treatment with TMR-Scrambled peptide did not induce contractions.

**Reference**

1. Holmes, S.P., He, H., Chen, A.C., Ivie, G.W., Pietrantonio, P.V. Cloning and transcriptional expression of a leucokinin‐like peptide receptor from the southern cattle tick, *Boophilus microplus* (Acari: Ixodidae). Insect Molecular Biology 9, 457-465 (2000).
